# Yellow Fever Inoculation

**Published:** 1886-01-20

**Authors:** 


YELLOW FEVER INOCULATION.—There is a bill before the United States Senate favoring the appointment of two yellow fever experts and a specialist in microscopy as a commission to visit Rio Janeiro to investigate the method of preventing the disease by inoculation. The bill ought to pass, and we suggest that Professor Gaston, of Atlanta, be placed on the commission. He resided for many years in Rio Janeiro ; understands the people and language, and is a man eminent in the profession.

